# A prognostic signature of pyroptosis-related lncRNAs verified in gastric cancer samples to predict the immunotherapy and chemotherapy drug sensitivity

**DOI:** 10.3389/fgene.2022.939439

**Published:** 2022-09-06

**Authors:** Yanan Wang, Xiaowei Chen, Fei Jiang, Yan Shen, Fujin Fang, Qiong Li, Chuanli Yang, Yu Dong, Xiaobing Shen

**Affiliations:** ^1^ Key Laboratory of Environmental Medicine Engineering, Ministry of Education, School of Public Health, Southeast University, Nanjing, China; ^2^ Department of Epidemiology and Health Statistics, School of Public Health, Southeast University, Nanjing, China

**Keywords:** gastric cancer, lncRNA, immunotherapy, TCGA, LASSO regression, pyroptosis, prognosis

## Abstract

**Background:** Pyroptosis is a recently identified mode of programmed inflammatory cell death that has remarkable implications for cancer development. lncRNAs can be involved in cellular regulation through various pathways and play a critical role in gastric cancer (GC). However, pyroptosis -related lncRNAs (PRlncRNAs) have been rarely studied in GC.

**Methods:** Pyroptosis-related gene were abstracted from the literature and GSEA Molecular Signatures data resource. PRlncRNAs were obtained using co-expression analysis. LASSO Cox regression assessment was employed to build a risk model. Kaplan-Meier (KM), univariate along with multivariate Cox regression analysis were adopted to verify the predictive efficiency of the risk model in terms of prognosis. qRT-PCR was adopted to validate the expression of PRlncRNAs in GC tissues. In addition, immune cell infiltration assessment and ESTIMATE score evaluation were adopted for assessing the relationship of the risk model with the tumor immune microenvironment (TME). Finally, immune checkpoint gene association analysis and chemotherapy drug sensitivity analysis were implemented to assess the worthiness of our risk model in immunotherapy and chemotherapy of GC.

**Results:** We identified 3 key PRlncRNAs (PVT1, CYMP-AS1 and AC017076.1) and testified the difference of their expression levels in GC tumor tissues and neighboring non-malignant tissues (*p* < 0.05). PRlncRNAs risk model was able to successfully estimate the prognosis of GC patients, and lower rate of survival was seen in the high-GC risk group relative to the low-GC risk group (*p* < 0.001). Other digestive system tumors such as pancreatic cancer further validated our risk model. There was a dramatic difference in TMB level between high-GC and low-GC risk groups (*p* < 0.001). Immune cell infiltration analysis and ESTIMATE score evaluation demonstrated that the risk model can be adopted as an indicator of TME status. Besides, the expressions of immunodetection site genes in different risk groups were remarkably different (CTLA-4 (r = −0.14, *p* = 0.010), VISTA (r = 0.15, *p* = 0.005), and B7-H3 (r = 0.14, *p* = 0.009)). PRlncRNAs risk model was able to effectively establish a connection with the sensitivity of chemotherapeutic agents.

**Conclusion:** The 3 PRlncRNAs identified in this study could be utilized to predict disease outcome in GC patients. It may also be a potential therapeutic target in GC therapy, including immunotherapy and chemotherapy.

## Introduction

Gastric cancer (GC) is a critical public health issue that should not be underestimated (Smyth et al., 2020). According to the latest estimates of the International Agency for Research on Cancer 2020, the number of new cases of GC reached 1,089,000 worldwide in 2020, and GC has become the fifth most frequent cancer and the fourth most frequent cause of cancer death globally, seriously threatening human health. There are many factors that affect the development of GC including *H. pylori* infection, age, gender, and dietary-behavior, etc., (Gonzalez et al., 2013; Oliveira et al., 2015; Praud et al., 2018; Kumar et al., 2020; Poorolajal et al., 2020). Therefore, there is still a long way to go in terms of GC prevention. Besides, the prognosis of GC, particularly at the advanced stage, is poor, and there is no reliable approach for estimating the prognosis of GC subjects.

Pyroptosis, which triggers strong inflammation by releasing dangerous molecules and inflammatory cytokines consisting of interleukin (IL) -18, IL-1β, etc., (Zhou and Fang, 2019), is a kind of necrotic and inflammatory programmed cell death resulting from facilitating caspase-1 activation (Broz and Dixit, 2016; Man et al., 2017; Rathinam et al., 2019; Wang et al., 2020). Pyroptosis is mainly mediated by inflammatory vesicles and excessive pyroptosis can lead to various inflammatory diseases. Pyroptosis and inflammation are important in mediating infectious diseases, immune disorders, etc. Numerous investigations in recent years have established that pyroptosis is remarkably linked to tumorigenesis (Ma et al., 2018; Wang et al., 2019; Zhou and Fang, 2019; Fang et al., 2020).

Long non-coding RNAs (lncRNAs) are a subclass of RNA molecules whose transcripts exceed 200 nucleotides in length (Ponting et al., 2009). Generally, they do not encode proteins, but can participate in protein-coding gene modulation as RNAs at various levels, consisting of epigenetic modulation, transcriptional modulation, and post-transcriptional modulation. Most lncRNAs have a conserved secondary structure, sheared form, and subcellular localization, which are important for lncRNAs to perform their functions. Although the majority of lncRNAs are expressed at a low level compared to messenger RNA (mRNA), many lncRNAs are of great importance in regulating cellular homeostasis and gene expression and have a central role in cellular processes, biological development and disease progression (Chen et al., 2018). Because lncRNA expression is very tissue-specific, it has the potential to be utilized as diagnostic along with prognostic biomarkers as well as therapeutic targets for some cancers (Deng et al., 2017; Chen et al., 2018; Li Y. et al., 2021).

Recently, it has been shown that lncRNAs can be involved in modulating the process and progress of GC. Some lncRNAs can promote cancer, and conversely, some lncRNAs can suppress cancer, but the detailed mechanism is not clear (Sun et al., 2016; Wei and Wang, 2017; Ren et al., 2020). Similarly, the role of pyroptosis in cancer also has some duality (Kolb et al., 2014). LncRNA plays an important role in pyroptosis, which can unbalance the inflammasome and lead to cell pyroptosis. It can also regulate pyroptosis through mediating different signaling pathways (Wan et al., 2020; Xu et al., 2020). Some studies have demonstrated the predictive value of pyroptosis-related long noncoding RNAs (PRlncRNAs) for the prognosis of cancer patients. This indicated a possible important role of PRlncRNAs in tumors (Lu et al., 2022). Given the limited amount of research on PRlncRNAs in GC, we started investigating whether PRlncRNAs may be employed as diagnostic, as well as prognostic indicators for the prevention along with treatment of GC.

Based on the TCGA database and quantitative real-time polymerase chain reaction (qRT-PCR), we screened for PRlncRNAs that play an important role in GC prognosis. We then constructed a risk model to further predict the prognosis of GC patients, and explored the predictive significance of the model in immunotherapy and chemotherapy, thereby providing a more reliable scientific basis for its use as a prognostic marker as well as an indicator of treatment response for GC patients.

## Materials and methods

### Data collection

**FIGURE 1:**
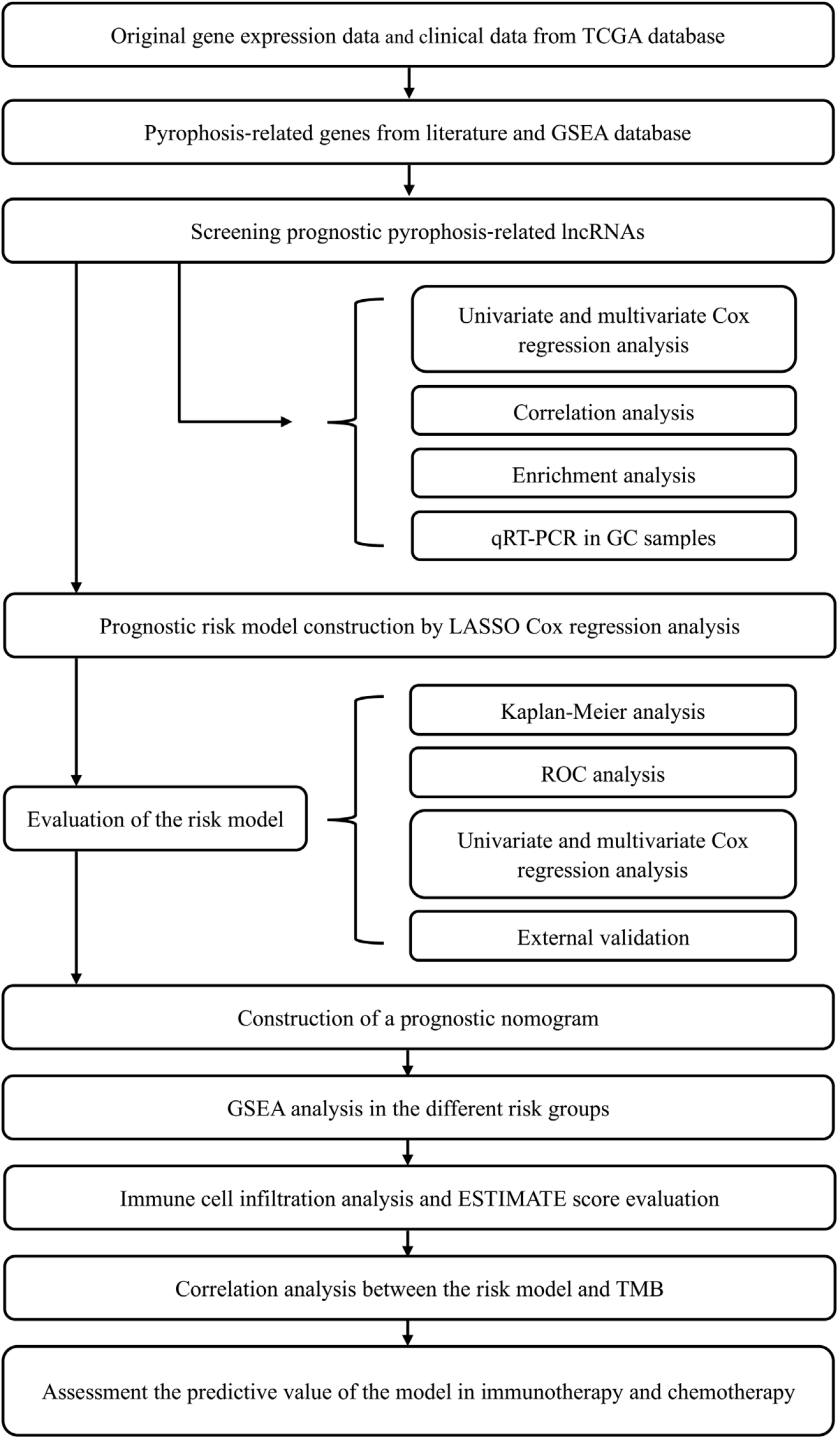
Flowchart of this study.

The original gene expression data (375 samples of GC tissues along with 32 samples of para-cancerous tissues) were obtained from the TCGA data resource (https://portal.gdc.cancer.gov). The original clinical data of the GC subjects were also abstracted from the TCGA data resource. Corresponding clinical information consisted of age, family history, gender, grade, pathological stage, along with vital status. The clinical characteristics of the subjects are shown in Supplementary Table S1. All our data were abstracted from TCGA, thus, approval from the Ethics Committee was not required. This research work was in full compliance with the guidelines for the NIH TCGA human subject protection and data access policies. The flowchart of this study was shown in Figure 1.

### PRlncRNAs co-expressed with pyrophosis-related encoding genes

A total of 50 pyrophosis-linked encoding genes (mRNAs) were abstracted from literature and the Molecular Signatures Database of Gene Set Enrichment Analysis (GSEA, http://www.gsea-msigdb.org/). Firstly, we screened out the differentially expressed lncRNAs in the tumor group and the adjacent normal group by the limma R package. Then co-expressed lncRNAs were assessed via creating a pyrophosis-linked mRNA-lncRNA co-expression network on the basis of the criteria of |Correlation Coefficient| > 0.4 and *p* < 0.001 through Pearson correlation analysis by the cor. test function. Finally, lncRNAs that are both differentially expressed and significantly co-expressed with pyroptosis-related genes are PRLncRNAs.

### LASSO cox regression analysis

Herein, the prognostic worthiness of these PRlncRNAs were screened by univariate along with multivariate Cox regression assessment firstly. Then, we created an efficient Risk Assessment Model by the least absolute shrinkage and selection operator (LASSO) Cox regression assessment via the glmnet package in R for modeling. We adopted the Glmnet package to explore the penalty parameter lambda through the cross-verification and uncovered the optimal lambda value. The optimal values of the penalty parameter were assessed by 1000-round cross-verification. We chose the most suitable lncRNA group to create a risk model. The median value of the risk score was adopted as the cut-off point. Herein, the patients were stratified into high-GC and low-GC risk groups. The risk score was calculated on the basis of a linear combination of the coefficients resulting from the LASSO regression model multiplied with the expression value of each selected lncRNA (coef: coefficient; expr: expression; lncRNAn: The n^th^ lncRNAs): Risk score = coef _(lncRNA1)_ *expr _(lncRNA1)_ +coef _(lncRNA2)_ *expr _(lncRNA2)_ +…+coef _(lncRNAn)_ *expr _(lncRNAn)_.

### Evaluation of the risk model

The area under the curve (AUC) for two-year, three-year, as well as five-year overall survival was estimated via the time-dependent receiver operating characteristic (ROC) curve, and the accuracy for survival estimation of the risk model was assessed with the survival package in R. To assess the effect of the risk model on patients’ rates of survival, we used univariate along with multivariate independent prognostic analysis.

We utilized independent prognostic criteria to create a prognosis nomogram via the R “rms” package in order to provide a quantitative tool for forecasting the rate of survival in the TCGA GC data set. Afterwards, a calibration curve was generated to check if the estimated survival outcome (two-year, three-year, and five-year survival) matched the observed outcome.

### GSEA analysis of prognostic lncRNAs and risk groups in the model

Gene Set Enrichment Analysis (GSEA) is a very powerful enrichment analysis method that can perform GSEA analysis against data from a variety of databases, including common Gene Ontology (GO) databases, Kyoto Encyclopedia of Genes and Genomes (KEGG) databases, etc., (Powers et al., 2018). Herein, we assessed the potential molecular mechanisms of prognostic lncRNAs, the cellular processes enriched in high-GC and low-GC risk groups on the basis of the KEGG library in GSEA software 4.1.0. The visualization of the results was carried out by R.

### Correlation analysis between the model and TMB

The total number of non-synonymous mutations in every coding region of the tumor genome was characterized as Tumor mutation load (TMB), which included the total number of gene coding errors, base substitution insertions, and deletions (Lv et al., 2020; Zhang et al., 2020). In this work, we abstracted the somatic mutation information via a Perl script, after which TMB value was determined via dividing the number of somatic mutations. R was utilized to merge the patient’s TMB information with the risk scores. We investigated the TMB levels of patients in different risk categories and the association between TMB and riskscore. Then we assessed the rates of survival of patients with varied TMB levels.

### Immune cell infiltration analysis and ESTIMATE score evaluation

Cell-type Identification by Estimating Relative Subsets of RNA Transcripts (CIBERSORT) is a deconvolving algorithm-based analytical resource for estimating the composition and number of immune cells in mixed cell (Newman et al., 2015). To accurately assess the composition of immune cells in the tumor microenvironment, we utilized the CIBERSORT algorithm to calculate and quantify tumor-infiltrating immune cells from RNA sequencing data to analyze whether different types of immune cells infiltrate differently in the high-GC risk and low-GC risk groups.

In this research work, the immunoscore for every patient was computed with the ESTIMATE approach utilizing the “estimate” R package. ESTIMATE is a popular enrichment algorithm, which was extensively utilized in medical studies (Liu et al., 2021a; Liu et al., 2021b; Liu et al., 2021c). The abundance ratio matrix of 22 immune cells for each sample was acquired by cell type identification by estimating relative sub-sets of RNA transcripts (CIBERSORT: https://cibersort.stanford.edu/). The algorithm of 1,000 permutations was employed. Only samples having a CIBERSORT p of < 0.05 were incorporated in the subsequent analysis of comparing differential immune invasion levels between the sub-groups categorized by risk scores.

### Correlation analysis between the model and immunotherapy

The immune checkpoint is a key regulator of the immune system’s ability to suppress or stimulate systemic function (Gibney et al., 2016; Topalian et al., 2016). Immune checkpoint blockade (ICB) therapy is used to unblock the suppressive effect of tumor cells on immune cells by blocking the interaction between immune checkpoint expressing tumor cells and immune cells, thus restoring effective T cell function (Pardoll, 2012; Patel and Minn, 2018; Wei et al., 2018). We adopted the limma package to analyze whether the expression of common immune checkpoint genes (CTLA-4, B7-H3, VISTA, PD1, PD-L1, etc.) differed in high-GC and low-GC risk group, and thus to assess the significance of this risk model in assessing the benefits of immunotherapy.

To further assess the relationship of the risk scores with clinical chemotherapy, we predicted the sensitivity of chemotherapeutic agents and analyzed the differences in chemotherapy drug sensitivity between high-GC and low-GC risk groups. We utilized the “pRRophetic” R package to predict the drugs’ half-maximal inhibitory concentration (IC50) on the basis of the Cancer Cell Line Encyclopedia (CCLE) by ridge regression.

### GC samples collection and quantitative real-time polymerase chain reaction

A total of 40 pairs of GC tissues and Para cancerous tissues were acquired from Zhongda Hospital, Southeast University, and authorized by the Ethics Committee of Zhongda Hospital, Southeast University. All subjects signed an informed consent form. All the tissues were collected following surgical excision from individuals who had never undergone prior radiotherapy or chemotherapy. Then, we stored these samples at −80°C for further use.

We extensively assessed the expression of predictive lncRNAs in GC tissues and neighboring non-tumorous tissues via qRT-PCR. Isolation of total RNA from GC tissues was done with the TRIzol reagent (Invitrogen, Carlsbad, United States). After that, generation of cDNA was done with the PrimeScriptTM RT reagent kit (TAKARA). The qPCR reaction constituted a 20 μL mixture, comprising diluent cDNA 1 μL, 2x RealStar Power SYBR Mixture (GenStar, China) 10 μL, DEPC water 8.2 μL, forward primers (FP) and reverse primers (RP) 0.4 μL, respectively. The reaction was carried on the StepOnePlus PCR System (Applied Biosystems, United States) for 40 cycles (95°C for 15 s, 60°C for 30 s, and 72°C for 30 s) following a 2 min pre-denaturation at 95°C. Relative transcript expression was computed with the 2^−ΔΔCt^ approach and standardized to GAPDH. All primers were synthesized by Sangon Biotech (Shanghai, China). The sequence of the primers is as follows: GAPDH FP: TCA​AGA​TCA​TTG​CTC​CTC​CTG​AG; RP: ACA​TCT​GCT​GGA​AGG​TGG​ACA, PVT1 FP: TCC​ACT​CAC​TTT​GGC​CTT​TC; RP: AGG​TGA​ACA​CAG​AGC​ACC​AA, CYMP-AS1 FP: GAG​GTG​GTC​CTG​AGG​TTC​AA; RP: ACC​TTT​GTC​GGT​GCT​AGT​GC, AC017076.1 FP: AAG​TTG​AGG​TGG​CCC​TGA​AT; RP: TTT​AGC​TCA​CAT​CTG​TCC​AGT​CA.

### Statistical analysis

All statistical analyses and visualization were impelemented in R software 4.1.1 (https://www.r-project.org/), including R packages limma, pheatmap, igraph, reshape2, ggpubr, glmnet, forestplot, survival, survminer, timeROC, rms, foreign, utils, org. Hs.eg.db, clusterProfiler, enrichplot, vioplot, ggExtra, plyr, grid, gridExtra, pRRophetic, etc. Wilcoxon test was adopted for the comparisons between two groups. Survival analysis was done with the survival along with survminer R packages. The limma package was adopted to estimate the mean and variance of gene expression, immune cell infiltration levels, drug sensitivity, etc. in different subgroups to perform a variance analysis through a linear model. The Kaplan Meier method and the log-rank test were adopted for survival analysis and survival distribution comparisons. Logistic LASSO regression, univariate along with the multivariate Cox regression analysis were implemented to screening for effective prognosis-linked genes. Forest map was drawn by the R language ggforest package. *p*-value less than 0.05 was regarded as statistical significance.

## Results

### Identification and screening of pyrophosis-related lncRNAs

**FIGURE 2:**
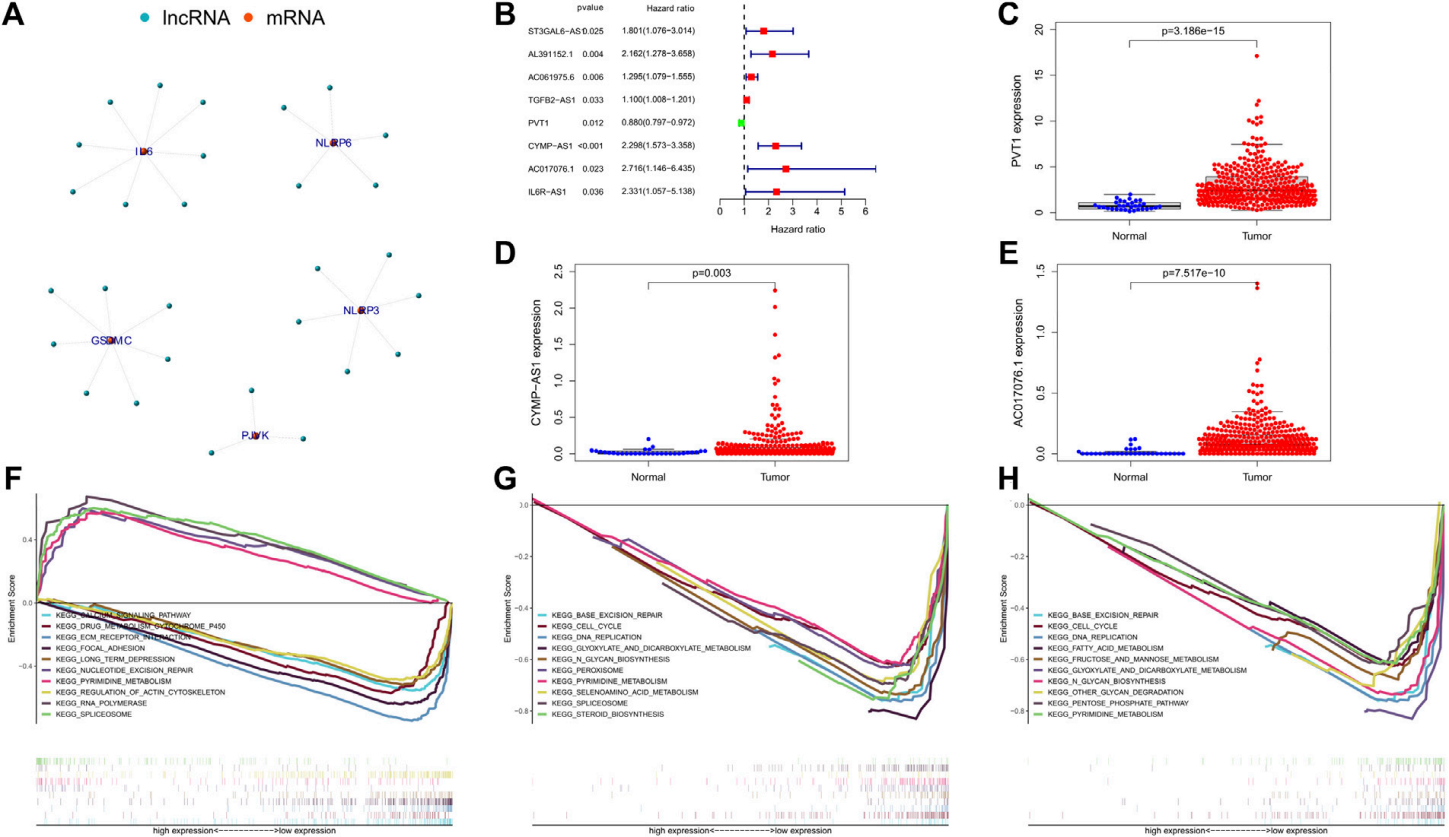
**(A)** Co-expression network of the pyroptosis-related mRNAs-lncRNAs was constructed and visualized using R. **(B)** The forest showed the HR (95% CI) and *p*-value of selected lncRNAs by univariate Cox regression analysis. **(C–E)** Visualization of the expression levels of lncRNAs with prognostic value expressed in human GC tumor tissues and adjacent normal tissues. **(F–H)** Represents the significantly enriched KEGG pathways of lncRNA PVT1, CYMP-AS1, and AC017076.1 in different expression level, respectively. GC, gastric cancer.

We have obtained 50 pyrophosis-related genes through searching literature and GSEA database (Supplementary Table S2). Based on the transcriptome dataset of GC cohort downloaded from TCGA database, we performed co-expressed analysis and constructed pyrophosis-related mRNA-lncRNA co-expression network. There were 29 lncRNAs strongly associated with pyrophosis-related genes (Figure 2A). Then, univariate Cox regression analysis was conducted on these 29 lncRNAs. A total of 8 lncRNAs were identified according to the criterion of *p* < 0.05 (Figure 2B). Subsequent multivariate Cox regression analysis indicated that only 3 lncRNAs (PVT1, *p* = 0.004; CYMP. AS1, *p* < 0.001; AC017076.1, *p* = 0.020) exhibited significant prognostic value for GC. In addition, the boxplot and heatmap showed that the expressions of these 3 lncRNAs were all higher in tumor samples than in adjacent normal samples (Figures 2C–E).

### Functional and pathway enrichment assessment of the 3 pyrophosis-related lncRNAs signature

To further clarify the possible biological processes involved in the 3 lncRNAs, we analyzed the pathways in which they were enriched by GSEA. The result showed that the low expression of PVT1 was mainly associated with these signaling pathways, including ECM receptor interaction, calcium signaling pathway, drug metabolism cytochrome p450, focal adhesion etc.; its high expression mainly focused on RNA polymerase, pyrimidine metabolism, spliceosome and so on (Figure 2F). Notably, CYMP. AS1 and AC017076.1 had no significant enrichment pathways corresponding to its high expression, while at low expression CYMP-AS1 affected N_glycan biosynthesis, base excision repair, selenoamino acid metabolism, pyrimidine metabolism, etc. (Figure 2G). Similarly, the low expression of AC017076.1 was related to these basic cellular metabolic processes (Figure 2H).

### The establishment of prognostic risk score model

**FIGURE 3:**
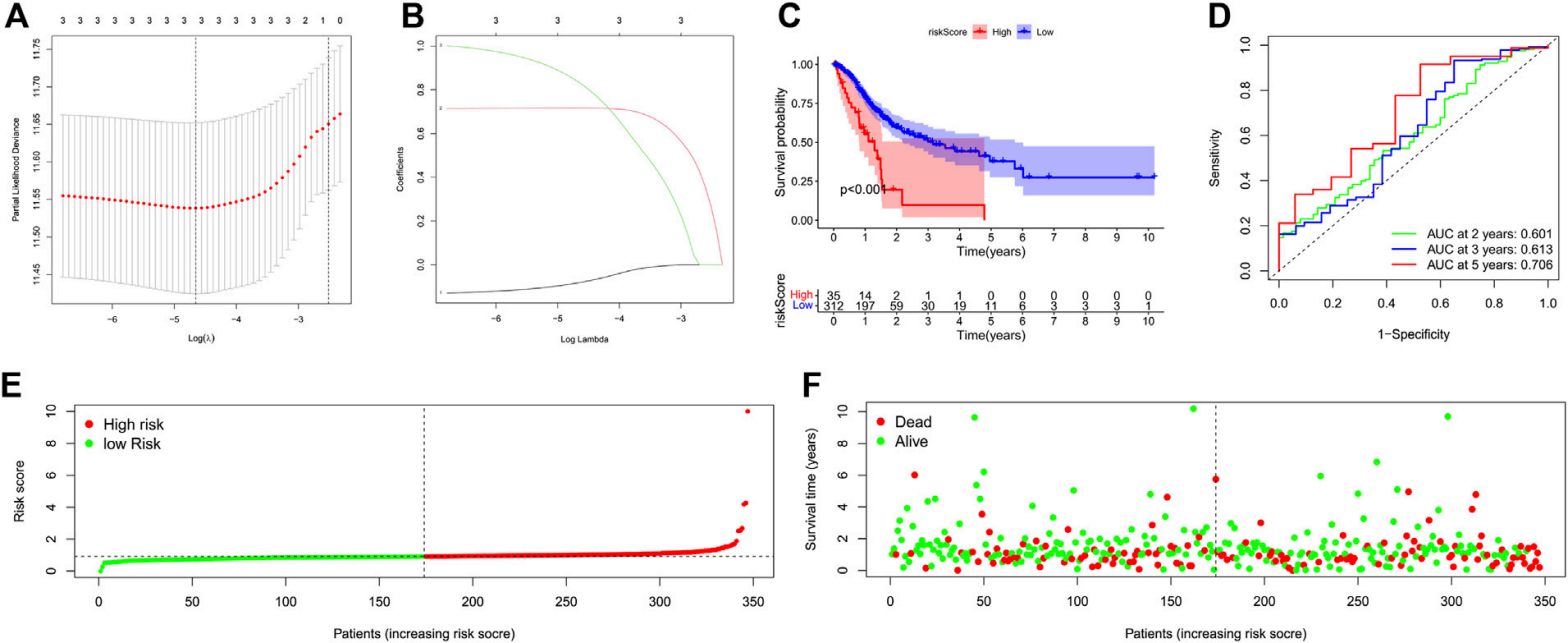
Construction of a 3-PRlncRNAs risk model and its predictive worthiness for GC subjects on the basis of TCGA data resource. **(A)** Logistic LASSO regression analisis on the optimum lncRNAs to create the final estimation model. The total number of lncRNAs is provided at the top of the figure. The deviation in partial likelihood is displayed versus log lambda. Dotted vertical lines designate the optimal values. **(B)** Profiles of the core lncRNA’s LASSO coefficients. The total number of lncRNAs is indicated at the top of the figure. Each curve corresponds to a certain key lncRNA, and the number next to it indicates the lncRNA’s serial number. **(C)** Kaplan-Meier survival analysis of the high-GC risk and low-GC risk groups based on the risk model and median risk score in GC patients. **(D)** The receiver operating characteristic (ROC) curve of the risk model for two-year, three-year, and five-year survival prediction. **(E)** The risk curve for each sample was on the basis of the risk score. **(F)** The scatterplot depicting each sample’s survival status. The green and red dots, respectively, signify survival and death. GC, gastric cancer; OS, overall survival. *p < 0.05; **p < 0.01; ***p < 0.001.

We applied Lasso Cox regression to the 3 lncRNAs and found they are all highly related to survival time of GC patients (Figures 3A,B). We calculated the risk scores of each GC patient with the LASSO Cox regression model based on the expression levels and the coefficients of these 3 lncRNAs. Risk score = (-0.0820120141988985*expression level of PVT1) + (0.717013150171401*expression level of CYMP-AS1) + (0.834152964623546*expression level of AC017076.1). According to the median risk score, All GC patients were divided into the high-risk (high risk score) or the low-risk (low risk score) group.

In addition, Kaplan-Meier survival curve was constructed to assess the associations between the expression levels of the 3-PRlncRNAs signature and overall survival (OS), As the Kaplan-Meier survival curve shows in Figure 3C, samples of high-risk group exhibited poorer OS than those of low-risk group (*p* < 0.001), suggesting that the prognostic signature of risk score is effective. Time dependent ROC analysis demonstrated that the prognostic accuracy of the 3-PRlncRNAs signature was 0.601 at 2-year, 0.613 at 3-year, and 0.706 at 5-year (Figure 3D). The risk curve and scatterplot were drawn to show the risk score and survival status of each GC patient. The risk coefficient and mortality of patients in the high-risk group were higher than those in the low-risk group (Figures 3E,F).

### Univariate and multivariate cox regression analyses of the prognostic ability of the risk model

**FIGURE 4:**
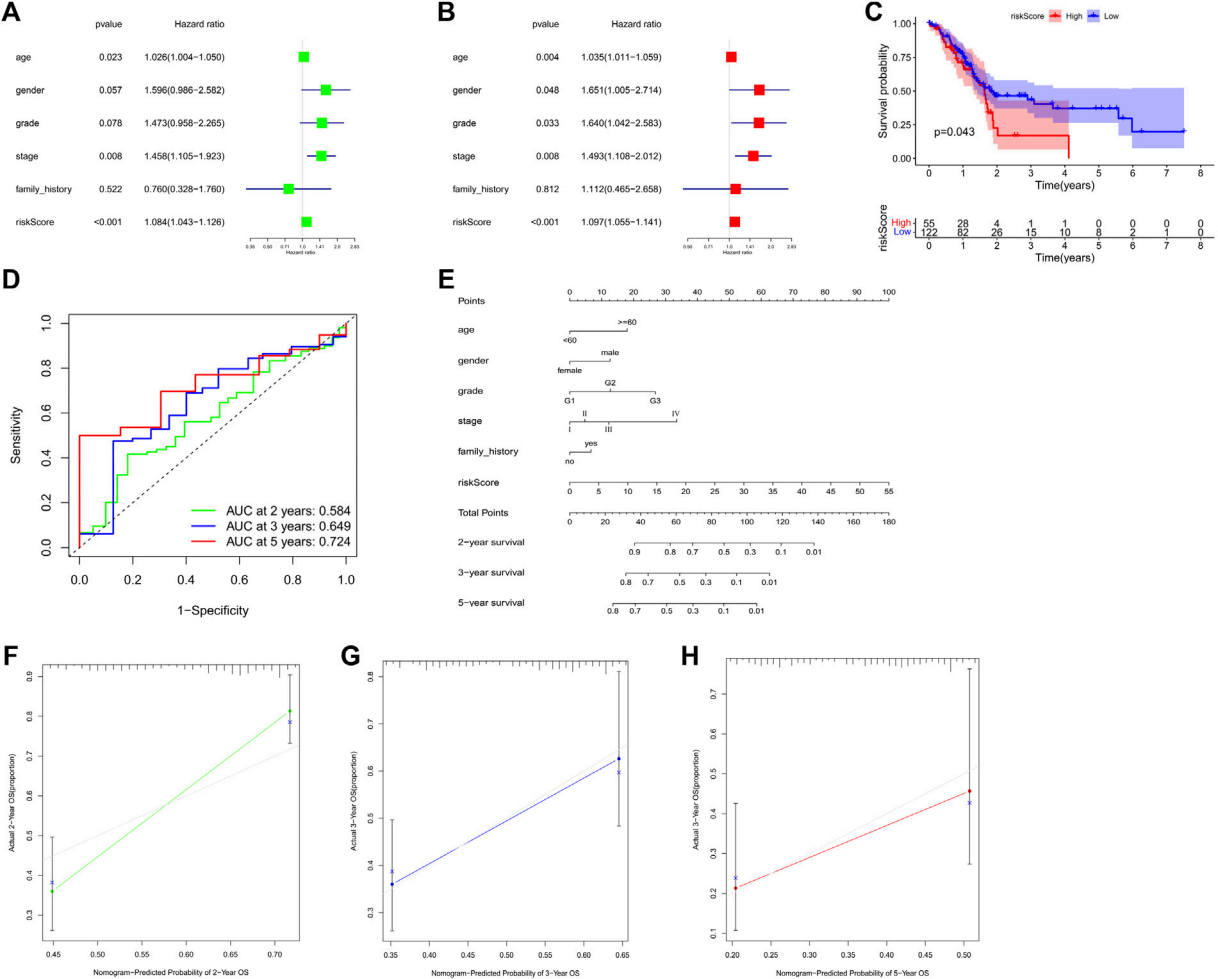
**(A,B)** Forest plot for the univariate **(A)** and multivariate **(B)** Model of the risk score along with clinicopathological variables on the basis of the Cox proportional hazard regression. Kaplan-Meier curve **(C)** and the receiver operating characteristic (ROC) curve **(D)** of the relationship between risk score and OS of PANC patients. **(E)** Nomogram for prognosis in subjects with GC based on risk score along with the clinical data. **(F–H)** The nomogram’s calibration curve. Perfect prediction is represented by a dashed line at 45°. GC, gastric cancer; PANC, pancreatic cancer; OS, overall survival. *p < 0.05; **p < 0.01; ***p < 0.001.

Univariate and multivariate Cox regression analysis were employed to estimate whether our model was a clinically independent prognostic factor for GC patients. The risk scores of the 3-PRlncRNAs signature and clinicopathological characteristics, including age, gender, grade, pathological tumor stage, were used as variables. Based on the GC cohort, univariate analysis indicated that the risk score (*p* < 0.001), age (*p* = 0.023), and pathological tumor stage (*p* = 0.008) were significantly associated with OS (Figure 4A). Subsequent multivariate analysis displayed that the risk score (*p* < 0.001), age (*p* = 0.004), pathological tumor stage (*p* = 0.008), gender (*p* = 0.048), and grade (*p* = 0.033) were significantly correlated with OS (Figure 4B). The results demonstrated that the risk score, pathological tumor stage and age were the optimal independent prognostic factors that could be used to predict the survival rate in GC patients. Especially, the prognostic 3-PRlncRNAs signature showed a higher significance in being an independent prognostic predictor for GC patients.

### External verification of the 3-PRlncRNAs signature in other cancers of digestive system

To estimate whether the prognostic 3-PRlncRNA signature had similar predictive values in different cohorts, we calculated the risk score for each sample according to the coefficients of these 3 PRlncRNAs to predict OS in other digestive system tumors from TCGA. A total of 177 pancreatic cancer (PC) patients were divided into a low-risk group and a high-risk group by the optimal cutoff value, and the OS of the PC patients in the low-risk group was significantly higher than that of the patients in the high-risk group (log-rank *p* < 0.05; Figure 4C). The 3-PRlncRNA signature constructed with the PC cohort also displayed a pretty accuracy in predicting the 2- year, 3- year, and 5-year OS, with AUC values of 0.584, 0.649 and 0.724 (Figure 4D).

### Construction of a nomogram for predicting survival

To offer a clinically applicable and quantitative tool for predicting the prognosis of GC patients, we further constructed a prognostic nomogram to predict the survival probability at 2-year, 3-year, and 5-year based on the TCGA GC cohort. Six independent prognostic parameters, including age, gender, grade, stage, family history and risk score, were enrolled in the prediction model (Figure 4E). The calibration curve of the prognostic nomogram showed good agreement between prediction and observation (Figures 4F–H).

### Functional and pathway enrichment assessment of high and low risk groups

**FIGURE 5:**
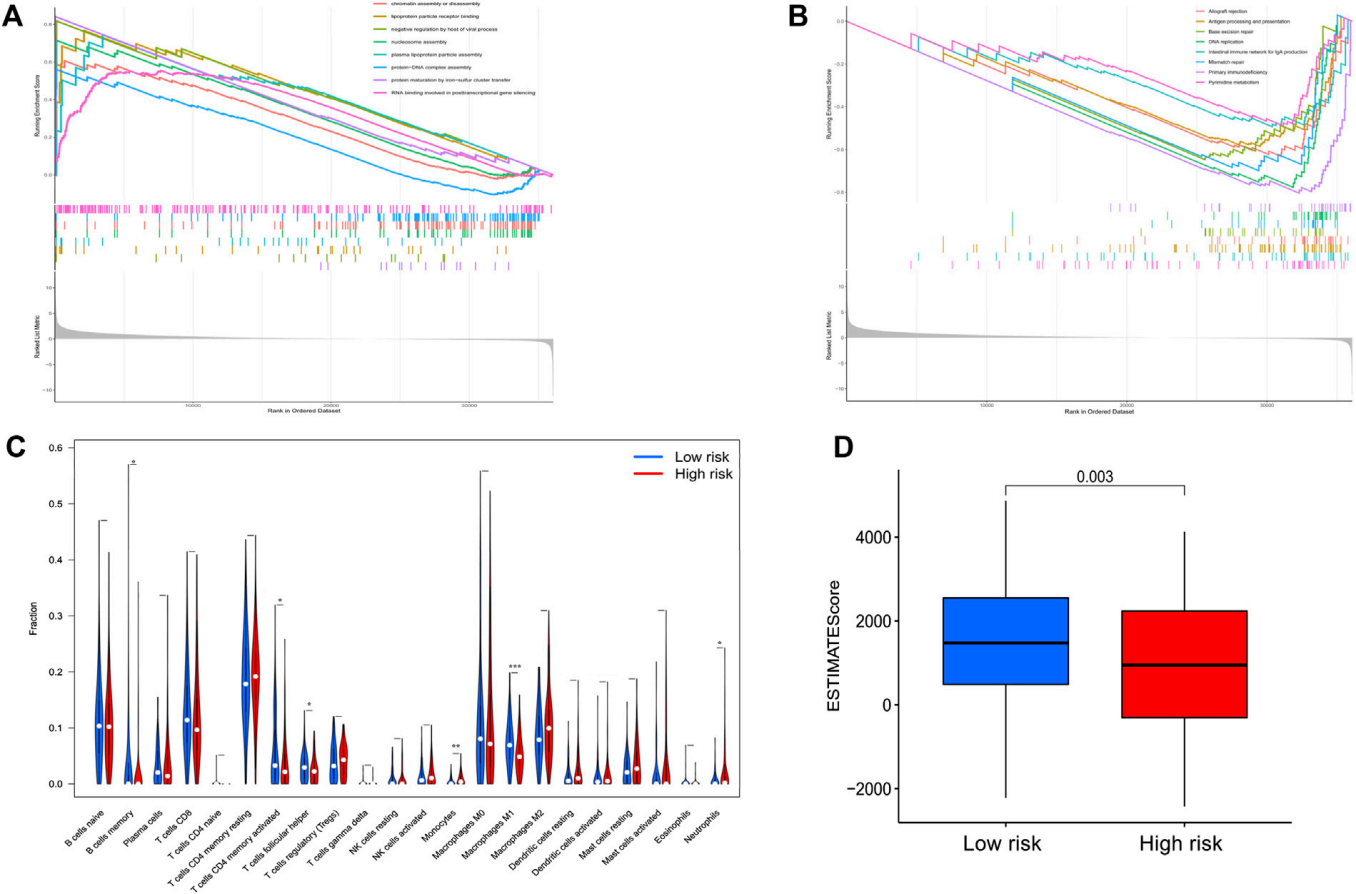
**(A,B)** Functional enrichment analysis of the two risk groups by GSEA. **(C,D)** Assessment of the correlation between the risk score of the GC patients and the complex immune infiltration level. **(C)** Violin plot displayed the distribution of diverse immune cell invasions in the high-GC risk and low-GC risk groups. **(D)** The ESTIMATE Score in different risk groups. The red group designates the high-GC risk group, whilst the blue group represents the low-GC risk group. GC, gastric cancer.

To investigate whether biological processes and pathways differed between the high and low risk groups, we performed GO and KEGG enrichment analysis. The results showed that the high and low risk groups exhibited differences in some basic cellular biological activities, including DNA replication, nucleotide metabolism, primary immunodeficiency, nucleosome assembly, protein−DNA complex assembly, etc. (Figures 5A,B).

### Correlation of the 3-PRlncRNA signature with immune cell infiltration

Considering the close relationship between pyroptosis and immunity, we explored the difference in immune cell infiltration between the two groups. Based on the ESTIMATE algorithm, we calculated the stromal score, immune score and ESTIMATEscore of each GC sample. Higher ESTIMATEscore (*p* = 0.003) were observed in the high-risk group compared with the low-risk group (Figure 5D), illustrating the different composition of tumor microenvironment in different risk groups. We further analyzed the abundance of 22 immune cells in the tumor microenvironment in the two groups. As the results shown in Figure 5C, in the high-risk group, the proportions of B cells memory (*p* = 0.044), T cells follicular helper (*p* = 0.013), Macrophages M1 (*p* < 0.001) and T cells CD4 memory activated (*p* = 0.019) were decreased, while the proportions of Monocytes (*p* = 0.006) and Neutrophils (*p* = 0.030) were increased compared with those in the low-risk group. High and low risk groups showed differential immune cells expression, which suggested that the 3-PRlncRNAs signature may be associated with prognosis by influencing the infiltration of immune cells in GC.

### Potential of the 3-PRlncRNAs signature as a predictor of response to immunotherapy

**FIGURE 6:**
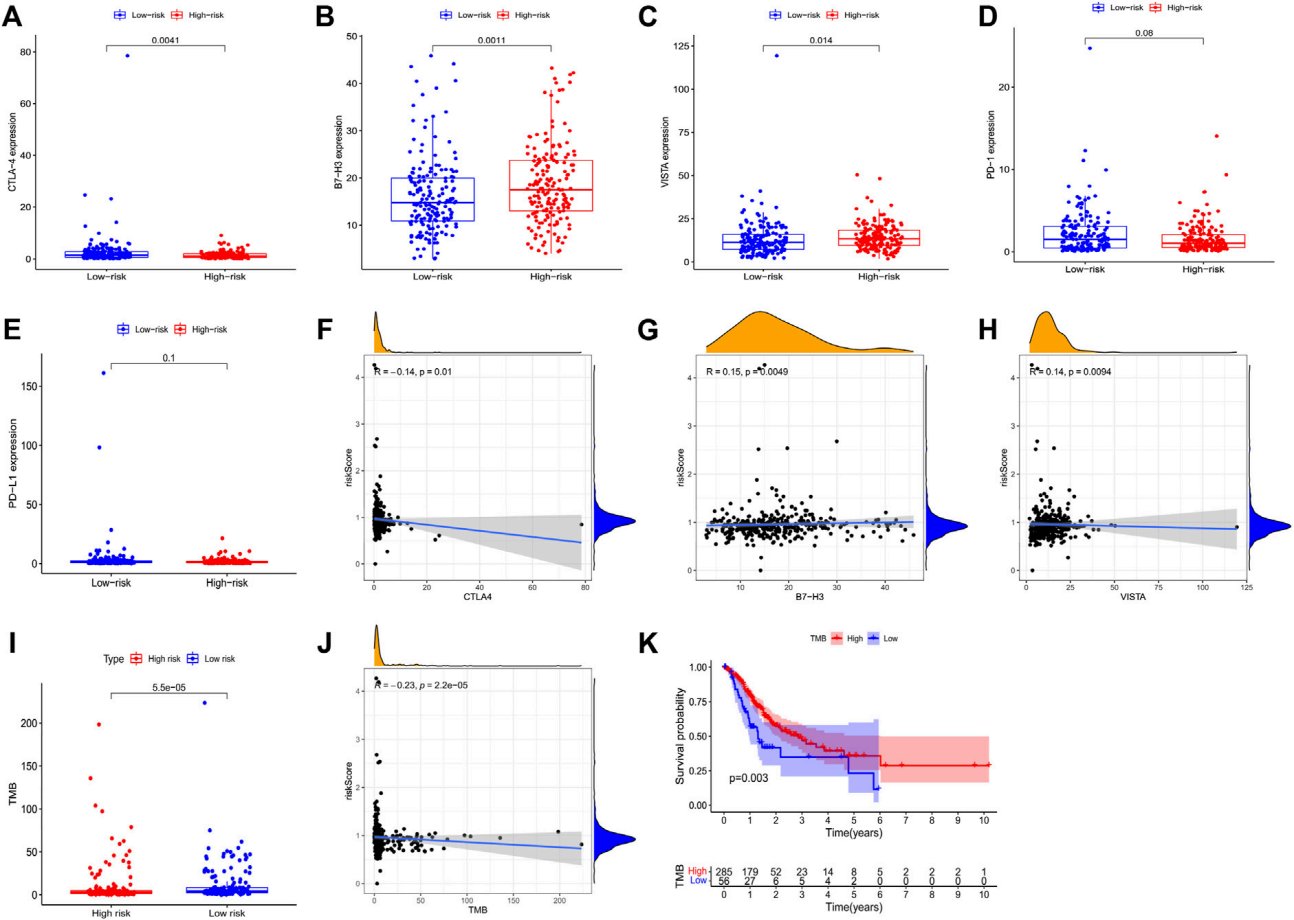
Correlation assessment of the immune checkpoint genes with GC patients’ risk score. **(A–E)** Exhibited the expression level of immune checkpoint genes in high- and low-GC risk groups. **(F–H)** Exhibited the correlation between the expression level of immune checkpoint genes and the risk score of GC patients. **(I)** A boxplot demonstrated the different TMB level in high-GC and low-GC risk groups. **(J)** The correlation of TMB with risk score. **(K)** Kaplan-Meier survival analysis of the high-GC and low-GC TMB groups in GC patients. GC, gastric cancer.

We selected six immune checkpoint genes that are clinically popular to assess the potential of risk models as indicators of immunotherapy response. The results showed that the risk score was significantly correlated with the expression of CTLA-4 (r = -0.140, *p* = 0.010), VISTA (r = 0.150, *p* = 0.005), and B7-H3 (r = 0.140, *p* = 0.009) (Figures 6A–H). These observed associations between our 3-PRlncRNAs signature and immunotherapy-related biomarkers indicated that GC patients in different group may have different sensitivity to immune checkpoint inhibitors.

### TMB was negatively associated with risk score and may predict patients’ survival probability

We analyzed the correlation of the 3-PRlncRNAs signature with TMB. Our result presented a markedly higher level of TMB in the low-risk group than the high-risk group (*p* < 0.001) (Figure 6I). Consistently, correlation analysis showed that patients with high TMB levels had lower risk scores than those with low TMB levels (r = -0.230, *p* < 0.001) (Figure 6J). Moreover, in Kaplan-Meier survival analysis, GC patients with high TMB levels had significantly higher survival rates than those with low TMB levels (*p* = 0.003) (Figure 6K).

### 3-PRlncRNAs signature was predictive to chemotherapy

**FIGURE 7:**
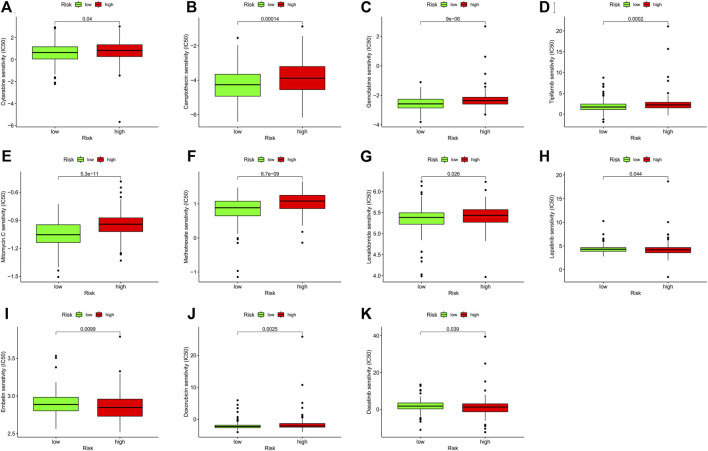
The correlation analysis of the sensitivity of chemotherapeutic agents with GC patients’ risk score. **(A–K)** Represented eleven chemotherapeutic agents’ IC50 in different risk groups. The green and red boxes represent low-GC risk and high-GC risk group, respectively. GC, gastric cancer.

In addition to exploring the relationship between risk models and immunotherapy, we further investigated whether risk models could be applied to the clinical use of drugs, especially chemotherapeutic drugs. Thus, we analyzed the differences in the sensitivity of ten chemotherapeutic agents, which have been widely used in the clinical treatment of tumors in recent years, in high and low risk groups. The results demonstrated a significant difference in the sensitivity of Tipifarnib (*p* < 0.001), Mitomycin (*p* < 0.001), Methotrexate (*p* < 0.001), Lenalidomide (*p* = 0.026), Lapatinib (*p* = 0.044), Embelin (*p* = 0.009), Doxorubicin (*p* = 0.003), Dasatinib (*p* = 0.039), Cytarabine (*p* = 0.040), Gemcitabine (*p* < 0.001) and Camptothecin (*p* < 0.001) in the high and low risk groups, which may be of critical use in the treatment of tumors, especially GC (Figures 7A–K).

### Quantitative real-time polymerase chain reaction of GC samples

**FIGURE 8:**
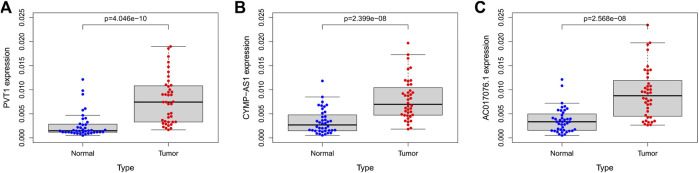
**(A–C)** The expression of lncRNA PVT1, CYMP-AS1, and AC017076.1 in tumor tissues and normal para cancerous tissues of GC patients, respectively. GC, gastric cancer.

We compared the expression levels of these 3 lncRNAs in 40 pairs of GC tumor tissue and normal para cancerous tissue samples. qRT-PCR was conducted to validate the expression level of these lncRNAs in frozen tissues. Expectedly, all the 3 lncRNAs were upregulated in GC tumor tissues than in normal para cancerous tissues (n = 40, PVT1, *p* < 0.001; CYMP. AS1, *p* < 0.001; AC017076.1, *p* < 0.001) (Figures 8A–C).

## Discussion

As one of the most frequent malignant tumors in the world, GC seriously affects people’s health. Although the growth in the incidence of GC has slowed in recent years, it still poses a significant disease burden, due in large part to the poor treatment outcomes as well as poor prognosis and low overall cure rate (Pinheiro et al., 2014; Luo et al., 2017; Machlowska et al., 2020; Sexton et al., 2020). Pyroptosis constitutes an inflammatory programmed cell death mediated by multiple inflammatory vesicles that play a pivotal role in a variety of diseases, for instance atherosclerosis (Xu et al., 2018), inflammation-related diseases (Crusz and Balkwill, 2015), tumors (Wang et al., 2019; Ruan et al., 2020), etc. . Some pyroptosis related genes, including the well-known gasdermin (GSDM), have been found can remarkably regulate the gastric carcinogenesis (Zhang et al., 2019; Li et al., 2020). lncRNAs have been widely studied in tumors, and different lncRNAs play different biological roles in tumors and can regulate its development and progression through multiple pathways (Sun et al., 2016; Wei and Wang, 2017; Wei et al., 2020). It has been shown that lncRNAs can mediate cellular pyroptosis through certain mechanisms and further act on cancer cells. Many investigations have been conducted to establish the signature of PRlncRNAs to predict the prognosis of tumor patients in breast (Lv et al., 2021; Ping et al., 2021), ovarian (Tan et al., 2021), melanoma (Wu L. et al., 2021), lung (Lin et al., 2021), endometrial (Chen et al., 2021), liver cancers (Wu Z. H. et al., 2021) etc., but they have rarely been found in GC. Therefore, we started to establish a validated PRlncRNAs biomarker to predict the survival status and treatment outcome of GC patients.

A total of 50 pyroptosis genes were obtained by reviewing literature and searching the GSEA pyroptosis gene set (Zhou and Fang, 2019; Ren et al., 2020; Xiang et al., 2021). Then we performed the co-expression correlation analysis of these 50 pyroptosis genes based on the TCGA GC transcriptome data, and after the strict screening criteria, we obtained PRlncRNAs significantly associated with pyroptosis related genes. Based on these PRlncRNAs and TCGA GC cohort, we built an effective risk model by logistic LASSO regression. Logistics LASSO regression is a technique for selecting variables while fitting a high-dimensional generalized linear model (Wang et al., 2007; Lee et al., 2016). It was undertaken to reduce the number of variables and effectively avoid overfitting, as well as to choose the most appropriate lncRNAs for modeling. Cross-verification was adopted to establish the ideal lambda value for the penalty parameter. We obtained a risk model for three PRlncRNAs by creating a penalty function via logistic LASSO regression (PVT1, CYMP-AS1, and AC017076.1). The model separated the GC cohort into high-GC and low-GC risk groups. The low-GC risk group had a much greater survival rate. Cox analysis, univariate along with multivariate, validated that the 3-PRlncRNAs risk model is an independent predictor of disease outcomes in GC subjects. It is of interest that there is already a study on the lasso model of PRlncRNAs construction (Xu et al., 2022). However, we found that the component lncRNAs of this model were different from the lncRNAs we used. Our obtained pyroptosis related genes were comprehensive and pyroptosis related lncRNAs were screened by stricter criteria. Furthermore, we did not exclude the GC data in the TCGA data resource to ensure the integrity and randomization of the clinical data as much as possible. What`s more, we examined the expression of prognostic lncRNAs in pairs of GC tissues from hospital using qRT-PCR to initially validate our model. In addition, we tried to find other tumor cohorts in the TCGA database to validate the reliability as well as the applicability of our risk model. PC, like GC, is a cancer of the digestive system. PC is severely lethal and poses a very high threat to human health (Morrison et al., 2018). Similarly, when we placed PC patients into our risk model, patients in the low-risk group showed a longer survival time. These consistent results further make our model more convincing.

Plasmacytoma variant translocation 1 (PVT1) is a common lncRNA located in a cancer-related region chr8q24.21 region, consisting of 1716 nucleotides (Lu et al., 2017; Onagoruwa et al., 2020). Many reports have confirmed that PVT1 plays an indispensable role in GC (Li et al., 2016; Xu et al., 2017). The expression level of PVT1 is remarkably elevated and may promote the proliferation as well as migration of GC cells by activating STAT3-mediated signaling pathways (Zhao et al., 2018; Niu et al., 2020). Herein, we established that PVT1 expression was remarkably increased in GC tissues, which is consistent with previous studies. Wu et al. demonstrated that CYMP-AS1 can be used as a biomarker for GC (Wu H. et al., 2021), which further makes our model more convincing. However, AC017076.1 was less studied. We found the potential of AC017076.1 as a survival signature by using lasso algorithm. The value of AC017076.1 as an indicator needs to be further explored and verified.

The tumor microenvironment (TME) is a complex and integrated system. It consists of tumor cells, the surrounding immune cells, inflammatory cells, stromal cells, nearby mesenchymal tissue, microvasculature, various cytokines, and chemokines (Lei et al., 2020). Tumors are remarkably linked to TME, which can influence its microenvironment through releasing cell signaling molecules, enhancing tumor angiogenesis, as well as inducing immune tolerance. Interestingly, immune cells infiltrating in the microenvironment can influence the development, growth and even progression of tumor (Cassim and Pouyssegur, 2019; Hinshaw and Shevde, 2019). Pyroptosis plays a pivotal role in TME and thus may affect tumor progression. Cytokines produced by pyroptosis can regulate immune cells and thus affect the immune system (Li L. et al., 2021). In GC, immune cell infiltration is also critical for tumor immune microenvironment (Perrone et al., 2008). Consistently, we found that in the GC cohort of TCGA, the infiltration of B cells memory, T cells CD4 memory activated, T cells follicular helper, Monocytes, Macrophages M1, and Neutrophils had significant differences in the high- and low-GC risk groups. The ESTIMATE algorithm was adopted to estimate the stromal score and immune score of tumor samples based on transcriptomic data. The stromal score along with immune score represented the abundance of stromal and immune cells, respectively. These two scores were summed to obtain the ESTIMATE score, which can be used to estimate tumor purity (Yoshihara et al., 2013). In our study, the ESTIMATE score was remarkably higher in the high-GC risk group than in the low-GC risk group, indicating that tumor purity was higher in the high-GC risk group.

Therapeutic strategies of tumor include traditional surgery, radiotherapy, and chemotherapy, as well as targeted therapy, tumor vaccine and immunotherapy, which have emerged in recent years (Smyth et al., 2020). Immunotherapy of tumor is a treatment method that applies immunological principles and methods to specifically remove tumor lesions and inhibit tumor growth by activating immune cells in the body and enhancing the body’s anti-tumor immune response. Immunotherapy can break the tumor immune tolerance and overcome the immune escape mechanism (Petitprez et al., 2020). In recent years, immunotherapy has shown great development potential in antitumor clinical applications and is gradually becoming the future direction of tumor therapy. Common immune checkpoint genes, including PD-1 (Peng et al., 2020), PD-L1 (Topalian et al., 2020), CTLA-4 (Rowshanravan et al., 2018), VISTA(Rowshanravan et al., 2018), and B7-H3 (Du et al., 2019), are targets of immune checkpoint inhibitors. They are widely used in antitumor therapy and have produced good clinical effects. In our study, the expression levels of CTLA-4, VISTA, and B7-H3 were significantly different in the high and low risk groups, suggesting that our risk model may be closely related to immunotherapy for GC, and that patients with high expression of immune checkpoint genes may be more sensitive to these checkpoint inhibitors. Studies have shown that the efficiency of mono-immunotherapy is just 15–20% (Gettinger et al., 2018). And the combination of immune checkpoint inhibitors is a trend in the future, as it is more effective in overcoming resistance to immunotherapy and significantly enhances efficacy (Pollack et al., 2018; Heinhuis et al., 2019). PD-1 and PD-L1 showed no statistical significance in the high and low risk groups. We built a hypothesis that potential synergistic effects may emerge when PD-1 and PD-L1 were combined with CTLA-4, VISTA, or B7-H3, etc. Certainly, this hypothesis requires more research to prove it.

TMB represents the total number of mutations per megabase (Mut/Mb) in DNA sequenced in a given cancer. TMB is an indicator of the efficacy of immunotherapy and higher TMB may be associated with better outcomes with immune checkpoint inhibitor therapy (Topalian et al., 2016; Chan et al., 2019; Negrao et al., 2021). Many studies have found that the expression of common immune checkpoint genes PD-1, PD-L1, and CTLA-4 is synchronized with TMB, and high PD-1 expression levels corresponds to high TMB (Cristescu et al., 2018; Hellmann et al., 2018; Samstein et al., 2019). Similarly, in this study, the expression level of TMB was consistent with that of CTLA-4, so we think that the level of TMB could be fully considered when designing immunotherapy, which may be more beneficial to improve the clinical outcome. Pyroptosis modulates immune cells in TME. LncRNAs can regulate immune genes and play important roles in immune cell growth, differentiation, migration, and immune responses. Both pyroptosis and lncRNAs have important effects on the immune microenvironment in tumors and may contribute to the effects of immunotherapy. Therefore, we tried to investigate whether immunotherapy-related PRlncRNAs could be linked to TMB. We found a significant difference of TMB in different risk groups, suggesting that the 3-PRlncRNAs model might be effective in identifying different levels of TMB. Then, we explored the correlation between TMB and risk scores, which were negatively correlated. TMB can be used as an indicator to predict survival rate of tumor patients, a higher TMB often predicts a better prognosis (Samstein et al., 2019). This may be due to the higher sensitivity of patients with high TMB to immunotherapy, which in turn improves prognosis. The strong link between TMB and immunotherapy and prognosis, once proven, will be very beneficial for clinical interventions outcomes and the OS of cancer patients. Our study initially verified this, but more in-depth theoretical and clinical studies are needed to confirm the feasibility of this conjecture.

We have already mentioned that chemotherapy is one of the most basic and traditional treatments for tumors, and it is widely used in clinical practice. However, there is a major problem of resistance in chemotherapeutic drugs, which makes the therapeutic effect much less effective (Wu et al., 2014; Dallavalle et al., 2020). We therefore analyzed the role of the risk model in differentiating chemosensitivity. The IC50 of several common chemotherapeutic agents showed a significant difference in different risk groups, including Camptothecin, Gemcitabine, Methotrexate, Mitomycin. C etc.

However, our study also has some limitations. Firstly, the clinical data downloaded from the TCGA database for GC patients was not perfect. For example, some clinical information had lots of censored values, which made our analysis possibly biased to some extent. Secondly, some crucial clinical information was not provided, especially treatment measures the patient has received, which is important to the prognosis of patients. Above all, most of our study is database mining and analysis, with only a few clinical samples to initially validate our results, we need more clinical prognostic data to support our conclusions.

## Conclusion

In conclusion, we obtained 29 lncRNAs co-expressed by 50 pyroptosis genes. Then by univariate and multivariate Cox regression analysis and lasso algorithm, we finally constructed a risk model of 3-PRlncRNAs, which can effectively predict the survival rate of GC patients. We constructed a prognostic Nomogram based on the 3-PRlncRNAs model and clinicopathological parameters, which provides an accurate and effective means to assess the prognosis of GC patients. In addition, the 3-PRlncRNAs model was expected to be an emerging tool for immunotherapy effect assessment, which will bring great benefits to individualized treatment and medical decision making. Although we applied qRT-PCR for preliminary validation, further studies are needed to explore the prognostic value of the 3-PRlncRNAs signature and to confirm our conclusions, as most of our study was based on bioinformatics analysis carried out on retrospective data.

## Data Availability

Publicly available datasets were analyzed in this study. This data can be found here: The Cancer Genome Atlas (TCGA) database (https://portal.gdc.cancer.gov).
