# Supplementary material for: A prognostic signature of pyroptosis-related lncRNAs verified in gastric cancer samples to predict the immunotherapy and chemotherapy drug sensitivity
Source: Front Genet. 2022 Sep 6;13:939439. doi: 10.3389/fgene.2022.939439 (PMC9485603; doi:10.3389/fgene.2022.939439)
Supplement: Supplementary file 1 [file Table1.docx]

**Supplement Table S1 Clinical pathological parameters of patients with GC**

| Characteristics | Variable | Patients (443) | Percentages (%) |
| --- | --- | --- | --- |
| Age | < 65 years | 185 | 41.76 |
|  | ≥ 65 years | 253 | 57.11 |
|  | Unknown | 5 | 1.13 |
| Gender | Male | 285 | 64.33 |
|  | Female | 158 | 35.67 |
| Pathological stage | Ⅰ | 59 | 13.32 |
|  | Ⅱ | 130 | 29.35 |
|  | Ⅲ | 183 | 41.31 |
|  | Ⅳ | 44 | 9.93 |
|  | Unknown | 27 | 6.09 |
| Vital status | Alive | 356 | 80.36 |
|  | Dead | 87 | 19.64 |
| Family history | Yes | 18 | 4.06 |
|  | No | 324 | 73.14 |
|  | Unknown | 101 | 22.80 |

*Note: GC, gastric cancer. Data are presented as number (%).*
