# Supplementary material for: A prognostic signature of pyroptosis-related lncRNAs verified in gastric cancer samples to predict the immunotherapy and chemotherapy drug sensitivity
Source: Front Genet. 2022 Sep 6;13:939439. doi: 10.3389/fgene.2022.939439 (PMC9485603; doi:10.3389/fgene.2022.939439)
Supplement: Supplementary file 2 [file Table2.docx]

**Supplement Table S2 Pyroptosis-Related genes and lncRNAs**

| Genes | LncRNAs | Genes | LncRNAs | Genes |
| --- | --- | --- | --- | --- |
| CASP6 | AC116351.2 | PLCG1 | AC007566.1 | CHMP4C |
| CASP8 | AC024560.1 | PRKACA | AC061975.6 | CHMP6 |
| CASP9 | LINC00624 | PYCARD | TGFB2-AS1 | CHMP7 |
| GPX4 | LINC02816 | SCAF11 | PVT1 | CYCS |
| GSDMA | AC036108.2 | TIRAP | LINC02253 | ELANE |
| GSDMB | AC007923.1 | TNF | LINC02526 | GSDMD |
| GSDMC | ST3GAL6-AS1 | BAK1 | AC000120.2 | GSDME |
| IL6 | OVAAL | BAX | AL691420.1 | GZMB |
| NLRC4 | AC015712.1 | CASP1 | CYMP-AS1 | HMGB1 |
| NLRP1 | AL391152.1 | CASP3 | AC017076.1 | IL18 |
| NLRP2 | LINC02254 | CASP4 | IL6R-AS1 | IL1A |
| NLRP3 | AC007368.1 | CASP5 | AF038458.2 | IL1B |
| NLRP6 | AC010271.2 | CHMP2A |  | IRF1 |
| NLRP7 | AP002478.1 | CHMP2B |  | IRF2 |
| NOD1 | AL137802.2 | CHMP3 |  | TP53 |
| NOD2 | LINC01511 | CHMP4A |  | TP63 |
| PJVK | USP12-AS2 | CHMP4B |  |  |
